# How the Depletion in Mineral Major Elements Affects Grapevine (*Vitis vinifera* L.) Primary Cell Wall

**DOI:** 10.3389/fpls.2017.01439

**Published:** 2017-08-21

**Authors:** Luís F. Goulao, João C. Fernandes, Sara Amâncio

**Affiliations:** Linking Landscape Environment Agriculture and Food (LEAF), Instituto Superior de Agronomia, Universidade de Lisboa Lisboa, Portugal

**Keywords:** biomechanical hotspots, cell wall epitopes, cellulose, gene expression, *Vitis vinifera* L., pectin (d)esterification, xyloglucan

## Abstract

The noteworthy fine remodeling that plant cell walls (CWs) undergo to adapt to developmental, physiological and environmental cues and the observation that its composition and dynamics differ between species represents an opportunity to couple crop species agronomic studies with research on CW modifications. *Vitis vinifera* is one of the most important crops from an economic point-of-view due to the high value of the fruit, predominantly for winemaking. The availability of some information related to this species’ CWs allows researching its responses to imposed conditions that affect the plant’s development. Mineral deficiency, in particular nitrogen, phosphorus, potassium and sulfur, strongly affects plant metabolism, reducing both growth and crop yield. Despite the importance of mineral nutrition in development, its influence on CW synthesis and modifications is still insufficiently documented. Addressing this knowledge gap, *V. vinifera* experimental models were used to study CW responses to imposed mineral depletion in unorganized (callus) and organized (shoots) tissues. The discussion of the obtained results is the main focus of this review. Callus and shoots submitted to mineral restriction are impaired in specific CW components, predominantly cellulose. Reorganization on structure and deposition of several other polymers, in particular the degree and pattern of pectin methyl-esterification and the amount of xyloglucan (XyG), arabinan and extensin, is also observed. In view of recently proposed CW models that consider biomechanical hotspots and direct linkages between pectins and XyG/cellulose, the outcome of these modifications in explaining maintenance of CW integrity through compensatory stiffening can be debated. Nutrient stresses do not affect evenly all tissues with undifferentiated callus tissues showing more pronounced responses, followed by shoot mature internodes, and then newly formed internodes. The impact of nitrogen depletion leads to more noticeable responses, supporting this nutrient’s primary role in plant development and metabolism. The consequential compensatory mechanisms highlight the pivotal role of CW in rearranging under environmental stresses.

## Introduction

Grapevine/wine, together with olive tree/olive oil and cork oak/cork, is one of the three outstanding Mediterranean agriculture dual systems. Grapevine (*Vitis vinifera* L.) is one of the most economically important fruit crops due to the high value of the fruit and its importance for winemaking. Understanding the biology and physiology underlying vine performance and how they can relate to cultural practices is mandatory to project strategies that maximize vineyard profits.

Growth of the canopy and plant organs calls our attention to the primary cell wall (CW) and its paradoxical features. CWs are load-bearing, extensible viscoelastic structures that surround the cells, acting as an “exoskeleton” that provides structural and mechanical support. Nevertheless expansion of growing cells encompasses simultaneously CW loosening and maintenance of enough strength to withstand internal high turgor forces. CWs play a crucial role in the regulation of both rate and direction of growth and determine plant cells and organs morphology (Fry, 1986; Chebli and Geitmann, 2017). The plant CW dynamic complex has further functions such as control of diffusion through the apoplast, signaling, regulation of cell-to-cell interactions, carbohydrate storage, or protection against biotic (Vorwerk et al., 2004; Bethke et al., 2016) and abiotic (Zhong and Ye, 2007; Tenhaken, 2015) stresses.

Abiotic stress refers to environmental conditions unfavorable for growth and proper development. In nature, plants are often exposed to several stress conditions at the same time (Mittler, 2006; Mittler and Blumwald, 2010). Atkinson and Urwin (2012) found evidence that plant responses to multiple environmental stresses are distinct from those triggered by individual stress factors and not just additive. Recent reviews by Le Gall et al. (2015) and Tenhaken (2015) focus on the effect of abiotic stresses on CW remodeling, quoting temperature (cold or heat), drought, flooding, osmotic stress and salinity, air pollutants, while boron deficiency is the only referred nutrient stress factor addressed (Tenhaken, 2015). Together with biotic stresses, the former abiotic conditions were reviewed by Houston et al. (2016). Particularly relevant, in the context of our review, are the implications that some abiotic stressors exert also in terms of interfering with plant mineral nutrition, which is our main focus. In fact, drought impairs nutrient uptake from soil solution, although not linearly. However, under sustainable viticulture practices, grapevine deficit irrigation and limited nitrogen (N) application can be used to control vegetative growth, canopy development, yield, and fruit composition (Keller, 2005).

It is well described that deficiencies in major minerals, nitrogen (N), phosphorus (P), potassium (K) and sulfur (S), strongly affect metabolism with subsequent impacts on plant growth, crop yield and nutritional value and quality of the agronomic product (Amtmann and Armengaud, 2009; Fernandes et al., 2009; Tschoep et al., 2009; Zhu et al., 2012). Unquestionably, mineral deficiency, with other abiotic stresses, challenges CW integrity and activates control mechanisms (Tenhaken, 2015). Examples of CW-related effects of limited mineral nutrient availability in the whole plant include reduced organ growth rates that can be explained by inhibition of cell expansion (Palmer et al., 1996) via reduction of CW plastic extensibility (Taylor et al., 1993; Snir and Neumann, 1997). Also at the individual cell level, decreased length and relative elongation rates have been reported, for instance, in *Lolium perenne* developing under phosphates limitation (Kavanová et al., 2006) and Arabidopsis growing under phosphate limitation (Balzergue et al., 2017). Concerning the later, P deficiency inhibited Arabidopsis root cell elongation according to a fine-tuned process that involves signaling regulation of malate efflux channel and ferroxidase genes. This inhibition is preceded by peroxidase-dependent CW stiffening of pre-elongated cells in a process controlled by Fe redox cycling, and coupled with callose accumulation in the root apex (Balzergue et al., 2017). Likewise, the deficiency in sulfur induces the accumulation of peptides related with *Chlamydomonas reinhardtii* CW structural protein synthesis (Takahashi et al., 2001).

In the primary CW, cellulose is identified as the main load-bearing polysaccharide. Cross-linking glycans, also referred to as hemicellulose polysaccharides, bond to cellulose (Cosgrove, 2005) to form an extensive framework. Hemicellulose polysaccharides are diverse and include xyloglucans (XyG), xylans and mannans (Liepman et al., 2007). Current models propose that XyG linkages are restricted to a minor component of the CW, closely intertwined with cellulose at limited sites, named “biomechanical hotspots,” providing selective targets for CW loosening (Park and Cosgrove, 2012, 2015). This network provides tensile strength to the CW and is embedded, with covalent linkages at specific regions, in a surrounding phase of hydrophilic gels constituted by pectic polysaccharides (Cosgrove, 2001; Popper and Fry, 2005; Zykwinska et al., 2007; Marcus et al., 2008) that determines the regulation of the hydration status and ion transport, the definition of the porosity and control of the wall permeability (Willats et al., 2001). These features are, in turn, defined by the chemical structure of these polymers, particularly the branching degree and pattern, namely the decoration with neutral sugars and the degree and pattern of methyl and acetyl esterification, which can ultimately lead to either stiffening or weakening the CW (Goulao, 2010). In addition to polysaccharides, a third network composed of structural glycoproteins further contributes to the biophysical properties of the primary CW and cell adhesion (Showalter, 1993; Cosgrove, 1997).

After the sequencing and public availability of the *V. vinifera* genome (Jaillon et al., 2007; Velasco et al., 2007), genomic resources for this species have proliferated, allowing large-scale profiling studies of gene expression (Grimplet et al., 2007; Zenoni et al., 2010) and proteomics (Sarry et al., 2004; Castro et al., 2005; Grimplet et al., 2007; Vincent et al., 2007; Jellouli et al., 2008; Giribaldi and Giuffrida, 2010) during development. In berries, such approaches allowed the identification of members of CW-related enzymes previously overlooked such as pectin methylesterase inhibitors (PMEIs) or cellulose synthases (CesAs) to be involved in development (DeLuc et al., 2007; Zenoni et al., 2010) and stress responses (Fernandes et al., 2015). In other species, modifications in gene expression involved in CW organization were reported in response to mineral deficiencies (Watanabe et al., 2010; Zeng et al., 2016).

The use of complementary experimental methodologies allowed drafting a map of *V. vinifera* CW responses to different forms of mineral –N, P and S– deficit, which will be described hereafter, under the main objective of this review. An integrated approach that included Fourier–Transform Infrared (FT–IR) spectroscopy coupled with chemometrics and polymer quantification, including Gas-Chromatography monosaccharide quantification of CW fractions, delivered a broad picture of the metabolic events triggered upon mineral depletion (Fernandes et al., 2013). *V. vinifera in muro* responses and specific CW component rearrangement after the depletion of individual major nutrients, were described by immunolocalization assays (Fernandes et al., 2016a) and complemented with qPCR mRNA quantification for selected genes (Fernandes et al., 2016b). Employing different biological systems, namely callus as a dedifferentiated system, and shoots, as a model for plant differentiated tissues, was instrumental in challenging current models of primary CW arrangements and dynamics in this economically valued species (Fernandes, 2015).

## Impairment in Cell Wall Polymers and Possible Compensatory Mechanisms

**FIGURE 1:**
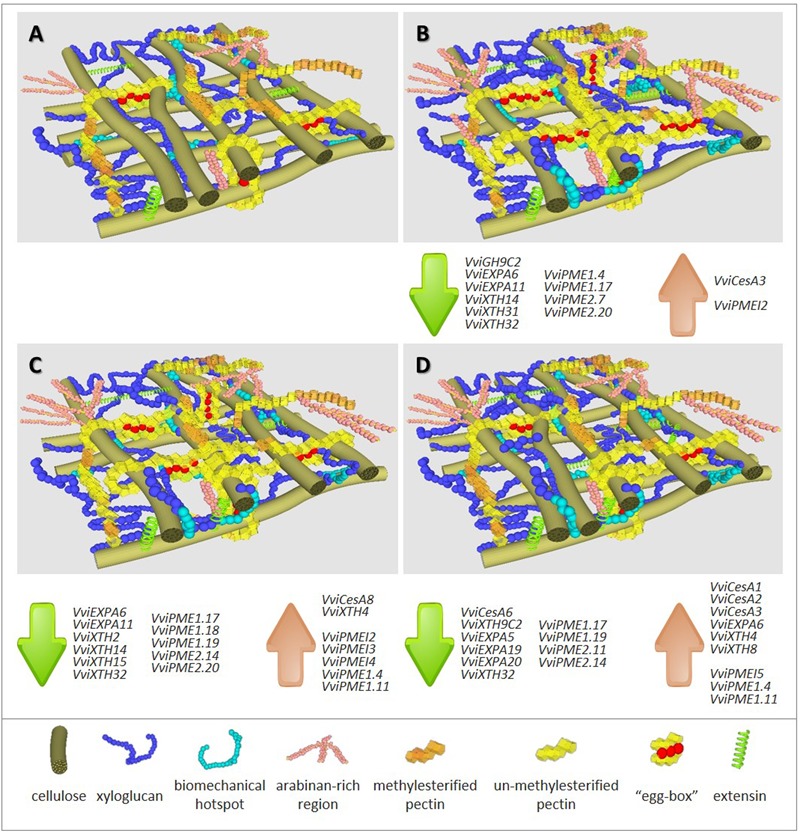
Representation of cell wall composition, architecture and expression trends of cell wall associated genes in grapevine biological systems under full major elements nutrition **(A)** and under depletion of nitrogen **(B)**, phosphorous **(C)** or sulfur **(D)**. Highlight summary of major changes in CW polymers abundance detected when compared with control conditions **(A)**, -N **(B)**: ▼▼▼ cellulose; ▲▲ xyloglucan; ▲ biomechanical hotspots; ▲▲ arabinan-rich regions; ▲▲ un-methylesterified pectins; ▲▲ calcium-linked pectin regions (“egg boxes”); ▲ extensins, -P **(C)**: ▼▼ cellulose; ▲ xyloglucan; ▲ biomechanical hotspots; ▲ arabinan-rich regions; ▲ un-methylesterified pectins; ▲ extensins, -S **(D)**: ▼ cellulose; ▲ xyloglucan; ▲ biomechanical hotspots; ▲ arabinan-rich regions; ▲ extensins. Gene accession numbers are given in Supplementary Table 1.

Global results using two model systems show that N and P deficiency brings about more pronounced responses than S shortage. Despite the observation of an equivalent trend, the extent of the responses depends on the individual plant tissue under stress. The common impacts of mineral deficiency on *V. vinifera* primary CWs include: (i) decreased cellulose content; (ii) increased total pectic polysaccharides that are rich in calcium-complexed homogalacturonic acid (HG) regions; (iii) increased pectin methylesterification; (iv) increased amounts of XyG and extensins. For each individual stress, observed changes are represented in **Figure 1** in which –N (panel **B**), –P (panel **C**) and –S (panel **D**) can be pair-wised compared with the control representation (panel **A**). A first map of CW modifications was obtained by FT-IR analyses in CW samples of homogeneous and reproducible callus growing in medium depleted in individual minerals (Fernandes et al., 2013). The negative values with respect to control observed between the peaks in wavenumbers 1033, 1041, and 1650 cm^-1^, assigned to C-OH groups of cellulose, to XyG and to proteins (Kačuráková et al., 2000), respectively, suggest a lower abundance of these components, most strikingly in –N samples. Conversely, the opposite trend was recorded for peaks in wavenumbers 950 and 1740 cm^-1^, related to a higher amount of pectin backbones (Coimbra et al., 1999) and pectin methyl-esters (Alonso-Simón et al., 2004), respectively.

These candidate CW modifications were confirmed using complementary quantification methods both in callus (Fernandes et al., 2013) and in two types of differentiated shoot tissues, the fourth (I-4) and the first (I-1) internode from the top of grapevine cuttings growing under the same mineral stress conditions (Fernandes et al., 2016a). The callus model allowed quantifying a reduction in cellulose content of ca. 43% under –N and ca. 12% under –P when compared to the control. In organized tissues, mineral deficiency impact on impairment of cellulose deposition occurs in both regions but was more pronounced in the older shoots. Fewer and longer internodes observed in –N I-4 shoots (Fernandes et al., 2016a), confirmed previous results in *Phaseolus* (Triplett et al., 1981), and the hypothesis that nitrogen deficiency induces changes in cell elongation and expansion (Cooke et al., 2003; Pitre et al., 2010; Plavcová et al., 2012).

Plants can compensate the impairment in biosynthesis or deposition of CW components by alternative mechanisms to keep the CW’s integrity (Pilling and Höfte, 2003; Wolf et al., 2012). Under –N and –P, both undifferentiated and differentiated tissues showed putative compensatory alternative mechanisms to less cellulose to reinforce CWs that included increased pectin content accompanied with a lower total degree of methylesterification and a higher proportion of arabinose-containing polysaccharides, probably tightly attached to the CW [**Figure 1**; compare panel **A** (control) with panels **B** (-N) and **C** (-P)]. A significant negative correlation between cellulose and pectin amounts was disclosed in the CW of the Arabidopsis mutant *kor1-1* (Nicol et al., 1998; Sato et al., 2001). In fact, the decrease in cellulose content due to a deficiency in an endo-1,4-beta-glucanase with a transmembrane domain, putatively involved in cellulose correct deposition, was accompanied by a 150% increase of pectin amounts (His et al., 2001), although the enzyme has no known association with the pectin content. Since pectins can function as mechanical tethers between cellulose microfibrils (Park and Cosgrove, 2012; Peaucelle et al., 2012; Wang et al., 2012) and long de-esterified stretches of HG acid favor the formation of calcium bridges, the proposed compensatory effect of reduced deposition of cellulose may result from the direct reinforcement of the glycan network and/or the stiffening or increased intercellular adhesion that outcomes from the formation of “egg-box” structures (Jarvis, 1984) and supramolecular pectic gels (Carpita and Gibeaut, 1993). This pattern of methyl de-esterification was confirmed by immunodot detection with the 2F4 antibody in *V. vinifera* callus and shoots CWs under –N (Fernandes et al., 2013, 2016a).

Complementary analyses employing *in muro* immunolocalization with specific monoclonal antibodies (mAbs) in the internodes system evidence a different arrangement of 1,5-arabinan side-chains in both I-1 and I-4. Highly heterogeneous rhamnogalacturonans-I (RG-I) are rich in neutral side chains (Caffall and Mohnen, 2009) and this diversity contributes to the definition of CW properties. Hence, the detection of high levels of arabinose may reflect an augmented biosynthesis of pectic polysaccharides or a higher side-chains substitution with arabinosyl residues. Under some abiotic stress conditions, RGI arabinosyl side chains can work as CW plasticizers (Harholt et al., 2010) supporting the suggested role of maintaining structural integrity. Ulvskov et al. (2005) analyzed the mechanical properties of *Solanum tuberosum* tuber CW in wild-type and transformed plants with decreased contents of arabinan and reported that the force which induces failure of the CW decreased in the latter. Further supporting the assumption of pectin compensatory role, the Arabidopsis cellulose synthase mutant *MUR10*/*CesA7* impaired in cellulose biosynthesis, showed an increase in pectic arabinan content (Bosca et al., 2006).

It is assumed that not all of the cellulose microfibril surfaces are covered with XyG (Bootten et al., 2004; Hanus and Mazeau, 2006; Park and Cosgrove, 2012). Nevertheless the CWs of an Arabidopsis XyG deficient mutant are more extensible than the wild-type (Park and Cosgrove, 2012). In conditions of impaired cellulose deposition, the amount and, most importantly, the XyG arrangement in the CW should act also as a compensatory mechanism to preserve the tissue’s integrity (Pilling and Höfte, 2003; Wolf et al., 2012). Regarding XyG, both I-1 and I-4 internodes showed immunolocalization of the LM15 mAb in the phloem, xylem, vascular bundle and parenchyma cells (Fernandes et al., 2016a). Under nitrogen deficiency significant amounts of XyG as well as more discrete points of accumulation were observed following a pattern that may be related with the “biomechanical hotspots” model (Park and Cosgrove, 2012, 2015).

In *V. vinifera* CWs, the effects of –S were less dramatic than –N and –P (symbolized in **Figure 1** comparing panel **A** with **D** vs. panel **A** with **B** or **C**). Although callus growth under –S was similar to that observed under –N, no significant reduction in cellulose was noticed (Fernandes et al., 2013). Nonetheless, in this condition, biological material, was more prone to the formation of egg-box structures, similarly to –N conditions. An increased deposition of extensin was observed, confirming the previous observations of this structural protein in response to several stresses (Showalter et al., 1992; Hirsinger et al., 1997; Ueda et al., 2007). Primary CW extensins (Showalter, 1993) have been implicated in the control of CW extension and strengthening by the formation of intermolecular peroxidase-mediated or covalent extensin-pectin cross-links (Qi et al., 1995; Jackson et al., 2001; Nuñez et al., 2009). The contribution of extensins to compensate the dramatic reduction in cellulose in –N growing callus also occurred in –S conditions [see **Figure 1** by paralleling panels **B** (–N) and **D** (–S) with control panel **A**]. Conversely, in organized tissues of internodes formed under –N and –P conditions tissues, the mAb reactive extensin epitopes (LM1) showed a lower signal was observed under –S environments.

## Biosynthesis and Remodeling Enzyme Activity and Gene Expression

Quantification of enzyme activity and expression of the respective coding genes have been useful research methods in addressing the regulation of synthesis and remodeling of plant CW polymers. CW-modifying enzymes exist in large multigene families (Farrokhi et al., 2006; Lerouxel et al., 2006), as result of more than 2000 genes (Carpita et al., 2001) that express according with distinct patterns among cells, tissues and development stages. Enzyme functions have been unveiled looking at genotypes impaired or overexpressing particular gene members (e.g., Osato et al., 2006; Peaucelle et al., 2008; Miedes et al., 2010). To our knowledge, at the molecular regulation level, no results of systematic research on the CW specific composition and arrangement modifications in response to –N, –P and –S deprivation are available.

High-throughput analysis allowed to identify CW genes responsive to phosphate starvation (Zeng et al., 2016) or sulfur depletion (Watanabe et al., 2010). To address the expression of candidate gene families, *V. vinifera* callus system was used as biological resource (Fernandes et al., 2016b). Most gene functional categories encompass overlapping multigenic families, and coding genes related to CW-biosynthesis and remodeling follow a pattern which may result in compensatory mechanisms to account for altered transcription (Giovannoni, 2004). The sequencing of the *V. vinifera* genome (Jaillon et al., 2007) and transcriptome (Velasco et al., 2007) allowed recovering the sequence of members of grapevine CW synthesis and remodeling multigene families. Mineral stress conditions gave rise to the expression of key candidate genes from all selected families (Fernandes et al., 2016b). Despite the more pronounced effect of –N in callus CWs (Fernandes et al., 2013), a higher number of genes with significant altered expression is observed after P and S imposed deficiency, suggesting complex regulation pathways (see affected genes listed associated to each representation in **Figures 1B–D**).

The decrease in cellulose content, the most striking result obtained in callus growing under –N and –P, (Fernandes et al., 2013) cannot be explained by a transcription impairment of *V. vinifera* cellulose synthase (*VviCesA*) genes. *CesA* multigene families encompass 18 members in *Populus trichocarpa* (Suzuki et al., 2006) and at least 10 members in *Arabidopsis thaliana, Oryza sativa* (Richmond and Somerville, 2000), and *V. vinifera* (Fernandes et al., 2016b). In *V. vinifera* callus, none of the CesA members was significantly down-regulated and –N and –P conditions, respectively, triggered the expression of *VviCesA3* and *VviCesA8.* Moreover, the expression of *VviCesA6* was inhibited in –S, (Fernandes et al., 2016b), interestingly, the only tested mineral that did not impact cellulose amounts (Fernandes et al., 2013).

In Arabidopsis mutants defective in CesA, a significant reduction in cellulose content and a moderate irregular growth were observed (Desprez et al., 2007; Handakumbura et al., 2013) confirming CesAs requirement for cellulose synthesis (Somerville, 2006; Endler and Persson, 2011). However, CesA activity is not enough for the formation of the cellulose fibril network, suggesting that correct cellulose synthesis, deposition or assemblage requires the coordinated contribution of other components (Takahashi et al., 2009). The sub-classes A and C of the glycosyl hydrolase 9 (GH9) family (beta-1,4-endo-glucanase) are known to participate in cellulose biosynthesis. Mutant backgrounds impaired on *KORRIGAN* (*KOR*), a GH9A expression (Urbanowicz et al., 2007a), show the requirement of KOR for the correct cellulose assemblage in elongating cells since the cells showed significantly reduced cellulose content even under normal CesA members expression (Nicol et al., 1998). It is interesting to note that KOR is reported as an integral part of the CesA complex (Vain et al., 2014). In *V. vinifera* callus mineral stress did not show significant modifications in the expression of CH9A family gene members, except for class C *VviGH9C2*, which was severely down-regulated under –N and, unexpectedly but confirming the *VviCesA6* expression, under –S conditions (Fernandes et al., 2016b). Class C GH9s hold a cellulose binding module (CBM) attached to the C-terminus of the catalytic domain (Urbanowicz et al., 2007b) and in *P. trichocarpa* it was demonstrated that *PtGH9C2* regulates cellulose crystallinity (Glass et al., 2015). It was proposed that cellulose crystallinity affects the expansion of the primary CW (Fujita et al., 2011) due to a lower number of potential cross-links with hemicelluloses (Lai-Kee-Him et al., 2002). The role of GH9C members in CW cellulose assemblage deserves a more detailed investigation.

Expansins (EXP) are directly implicated in changes in CW extensibility and strength and associated to lower levels of cellulose. These CW remodeling proteins reversibly break up hydrogen bonds between glycan groups from cellulose microfibrils and matrix XyGs as demonstrated by *in vitro* CW loosening (McQueen-Mason and Cosgrove, 1995; Cosgrove, 2000; Whitney et al., 2000; Wang et al., 2013). The expansin gene family encompass α-expansin (EXPA), β-expansins (EXPBs), expansin-like A (EXLA), and expansin-like B (EXLB) subfamilies. The association of EXPAs to cell expansion and plant growth and development (Cosgrove, 1999, 2000), was confirmed by studies using transgenic backgrounds (Choi et al., 2006). In *V. vinifera, VviEXP6* and *VviEXP11* were down-regulated in response to N and P deficiencies, more severely in –P conditions. Assuming the decreased cellulose content, the combination of more biomechanical hotspots with a down-regulation of EXPA genes may explain a mechanism to hold CW loosening, preserving its integrity. Conversely, S starvation, which does not impair cellulose deposition, led to the repression of *VviEXPA5, Vvi-EXPA19* and *VviEXPA20* transcripts together with the activation of *VviEXP6*, again a surprising result in line with the down-regulation of cellulose biosynthesis genes.

Xyloglucan endotransglycosylases/hydrolase (XTH) enzyme family encompasses members that induce CW extensibility in *in vitro* assays (Van Sandt et al., 2007). Out of the 10 XTH members expressing in *V. vinifera* callus tissues, sequence analyzes of two members, *VviXTH31* and *VviXTH32*, predicted hydrolase activity (Eklöf and Brumer, 2010). The expression of both transcripts was altered by mineral stress. The latter is repressed in all stress conditions while the former was down-regulated only under –N. The expression pattern of the remaining XTHs was dependent of the absent mineral. Similarly to EXPAs, a trend for a down-regulation under –N and –P was observed, while under –S an up-regulation pattern was quantified in two members and only a single member was repressed (Fernandes et al., 2016a). XTHs action can promote polysaccharide chains elongation independently from activated nucleotide sugars, so the involvement of some members in the formation of covalent linkages between cellulose microfibrils can also be proposed (Shinohara et al., 2017). Enzyme activity detected in extracts of plant elongation zones such as tomato hypocotyls or Arabidopsis roots over- or down-expressing/silenced members of the XTH family agree with changes in gene expression and correlate positively with CW extensibility and organ growth (Osato et al., 2006; Miedes et al., 2010; Wilson et al., 2015). Conversely, the effect on growth patterns or developmental phenotypes of some XTH isoforms is negligible (Kaewthai et al., 2013), suggesting their role in a XyG-recycling pathway. This pathway could modulate reinforcement in the XyG network due to the quantity of available non-reducing ends, prone to an increase in “biomechanical hotspots” (Sampedro et al., 2010, 2012).

Models of CW architecture were revisited after the empirical confirmation of covalent bonds between pectin polymers and cellulose microfibrils (Zykwinska et al., 2005, 2007; Park and Cosgrove, 2015) or matrix- glycans (Popper and Fry, 2005; Marcus et al., 2008). The new models highlight pectin methylesterases (PMEs) and their specific inhibitors (PMEI) (Bellincampi et al., 2004; Di Matteo et al., 2005; Juge, 2006; Jolie et al., 2010; reviewed by Goulao, 2010), which are known to significantly hit biophysical properties in the remodeling of CWs. In fact, the more pectin polymers host methyl groups the less hydrated calcium-linked gel structures are formed and the less can be depolymerized by polygalacturonase (PG) or pectate lyase (PL) hydrolysis. Both events contribute to increased wall stiffness and reduced creep and CW relaxation (Brummell and Harpster, 2001; Willats et al., 2001; Wakabayashi et al., 2003). The function and contribution of PMEs to CW loosening was experimentally proven, for instance, by over-expressing a PME in Arabidopsis which gave rise to a lower pectin methylesterification (Peaucelle et al., 2008; Hongo et al., 2012), or cell elongation inhibition (Derbyshire et al., 2007). Furthermore, the former effects could be reverted by overexpressing a member of the PMEI family (Peaucelle et al., 2011) and an equivalent equilibrium between PME and PMEI controlling the de-esterification rate was detected in Arabidopsis roots (Wilson et al., 2015).

As referred for other remodeling enzymes, PME genes express as a multigene family (Markovic and Janecek, 2004). Depending on the presence of a N-terminal PRO extension preceding the conserved PMEI domain (Jolie et al., 2010), PMEs are classified into two subfamilies: type I/group2 or type II/group 1 (Micheli, 2001). It is referred that group2 plant PMEs, with an acidic pI, act in a non-block-wise fashion, while group 1 plant basic pI PMEs, which represent the majority of the isoforms responding to mineral stress, act in a block-wise fashion (Bosch and Hepler, 2005). They produce long linear stretches of unesterified HG chains and promote Ca^2+^-linked gel structures formation and consequently, stiffen the CW. Coping with this model, basic PMEs represent solid candidates to act in compensatory mechanisms after the lack of cellulose. However, in the callus system model under –N, –P or –S conditions, with the exception of the increased expression of basic *VViPME1.4* and *VViPME1.11* under –P and –S, PME genes encompassing members from both groups were unaffected or down-regulated. Both transcripts are predicted to encode basic pI PMEs and the hypothesis of a significant role in the formation of “egg-boxes” should not be ruled out.

Interestingly, under mineral depletion, the expression of PMEI genes is globally up-regulated or not affected, again with different members responding to the individual mineral causing the stress. As a whole, an inhibition of PME gene expression upon mineral stress suggests a reduction in enzymatic de-esterification potential. Hence, the lower global degree (Fernandes et al., 2013) and the pattern of methylesterification (Fernandes et al., 2016a) that results from some mineral starvation conditions cannot be fully explained by the PME gene expression levels.

Four genes were affected by all individual stress conditions, namely *VViEXPA6, VviXTH32, VviPME1.4* and *VviPME1.17*. Interestingly, opposite trends of gene expression were observed in *VviEXPA6* for –S samples and *VviPME1.4* for –N material, when compared to the remaining two conditions. These findings suggest distinct regulatory pathways for individual mineral stresses, although pointing to the same goal of compensatory stiffening of the CW in result of deposition impairment of a given component.

Additionally to gene expression, the potential loosening activity of salt extracts from *V. vinifera* callus growing under N, P or S deficiency and the *in vivo* mechanical modifications that those extracts produce on dicot CW specimens was investigated. This model showed that under –N conditions, callus salt extracts have significantly inhibited total endoglucanase activity (Fernandes et al., 2016b). This result that putatively reflects an impairment in crystallization of the callus cellulose microfibrils and the associated CW reinforcement by more cellulose-XyG cross-links (Fujita et al., 2011), relates to enzymes with distinct activities. They may include endoglucanase, xyloglucan endotransglucosylase (XET), xyloglucan endohydrolase (XEH) or all. When applied to cucumber hypocotyls in extensibility tests, protein salt extracts from –N and –P callus promoted plastic deformation with no alteration in the elastic deformation (Fernandes et al., 2016b). This result sounds with the effects in CW stiffening considering that additional stiffening occurs in the absence of CW elastic deformation, including under abiotic stress (Micheli, 2001). On the other hand, plastic deformation is compulsory for the wall-loosening reactions involved in irreversible growth (Hohl and Schopfer, 1992). Since under –N and –P, callus produced increased extractable plastic deformation activity, this result may be related with the occurrence of CW stiffening, independent from growth regulation. Expansins and XTHs are CW-loosening agents that regulate CW expansion and cell enlargement through elastic deformation (Cosgrove, 2000; Miedes et al., 2013), therefore, the results obtained in extensibility tests sound with the general down-regulation of expansins and most XTHs genes quantified in response to mineral stress.

## Final Considerations

In general, abiotic stress refers to conditions that are unfavorable for plant growth. Mineral stress and drought, which are likely to affect the plant development, yield and, ultimately, the quality of the economic products, are included in the referred conditions.

Plant systems in controlled conditions are useful tools to assess stressful situations. The effect of mineral stress, N, P and S, could be investigated withdrawing individual minerals from *V. vinifera* growing substrates in two model systems. *V. vinifera* callus, as a dedifferentiated system, provided a useful model to understand CW responses to stress effects caused by mineral deficit, reducing eventual interference with other concomitant effects. Plant differentiated tissues, namely shoots as a more complex experimental system, complement the indications delivered by the simple callus model, since the organization of polymers in the CW is a key-factor for tissue organization.

The results highlighted in this review propose that *V. vinifera* responses to mineral stress include triggering compensatory mechanisms to keep CW integrity that would be compromised by the resulting reduction in the amounts of major load-bearing polymers. These observations also allow debating previous and current plant primary CW models. In fact, our results are better explained assuming the recently revised model that suggests load-bearing XyGs hidden in tight junctions that bind microfibrils together, instead of tethering cellulose microfibrils, and the resurgence of the interest in pectins composition and matrix–cellulose interactions in the CW structure.

Globally, the significant reduction in cellulose content observed under –N and –P suggests a specific controlled and fine-tuned sensing mechanism to sustain CW integrity. These findings are in accordance with Pilling and Höfte (2003) and Wolf et al. (2012) reports on the trigger of compensatory alternative reinforcement mechanisms via biosynthesis of novel material or establishment of new linkages when plant CW integrity is compromised. XyG may play an important role in the wall and influence its characteristics. Under nitrogen deficiency, the increased amounts of XyG can then be explained as a CW reinforcement mechanism, via biosynthesis of new material or establishment of new linkages. The reduced degree of pectin methyl esterification that occurred under mineral deficiency, specially –N, can be another response to low levels of cellulose. If the reduction of methyl esterification occurs in long stretches of HG chains, it promotes the formation of calcium bridges, the “egg–box” structures (Pelloux et al., 2007) that were labeled in most of the stress conditions. On the other hand, a higher substitution by arabinosyl residues in RGI side chains can work as plasticizers in CWs that undergo large physical remodeling (Harholt et al., 2010). Low levels of cellulose and high levels of arabinan enriched pectins were highlighted as a response to abiotic stresses (Le Gall et al., 2015).

Molecular evidences of CesA family gene expression fail to explain the observed reduction in cellulose content and drive the attention to members of other families, such as class C GH9s (Urbanowicz et al., 2007b), which hold predicted hydrolytic activity. Highlighted is the evidence that VvGH9C2 can be severely down-regulated under mineral stress. Similarly, the lower global degree and the pattern of methyl-esterification observed under some mineral starvation conditions cannot be directly explained by the inhibition PME genes. The exception may be the basic group 1 PMEs in –P and –S which act upon the HG chain in a linearly way promoting the formation of Ca^2+^-linked gel structures and, in this way, stiffening and reducing CW extensibility (Micheli, 2001). Furthermore, the observed up-regulated PMEI genes may regulate the activity of PMEs. One can also speculate that CW-related genes may function redundantly with the repression of one isoform compensated by the up-transcription of others (Matsui et al., 2005; Iurlaro et al., 2016).

A reliable picture of CW composition as well as tentative models of CW structure are available since last century 60–70 decades (Albersheim et al., 1967). The “tethered network” was the most consensual primary dicot CW model. In this representation, the hemicellulose polymers extensively link cellulose through hydrogen bonds to produce a load bearing tether, interleaved in an amorphous cement-like pectin matrix (Cosgrove, 2001). In this model, cellulose is described as the main load-bearing polysaccharide. However, the use of more sophisticated techniques allowed the attempts for a clear-cut model explaining CW structure and architectural organization (Cosgrove and Jarvis, 2012) and subsequent results disclosed the presence of covalent linkages between RGI-arabinan side-chains and cellulose microfibrils (Zykwinska et al., 2007) and between xyloglucan and pectins (Popper and Fry, 2005; Marcus et al., 2008) providing structural links between two major CW domains. Moreover, in agreement with the “biomechanical hotspot” concept (Park and Cosgrove, 2012; Peaucelle et al., 2012; Wang et al., 2012), not all of the cellulose microfibril surfaces are covered with XyG (Bootten et al., 2004; Hanus and Mazeau, 2006; Park and Cosgrove, 2012), so it is not surprising that other linkages, such as the referred cellulose and hemicellulose bonds with pectic polysaccharides are expected to maintain CW structure.

Assuming that only a limited cellulose surface is in contact with XyG (Bootten et al., 2004; Dick-Perez et al., 2011), pectins can be looked as hydrophilic dynamic polymers which may ease microfibrils motion as CW expands (Park and Cosgrove, 2015). Alternatively to XyG, pectic monosaccharides such as RG-I sidechains arabinan and galactan rich (Zykwinska et al., 2007, 2008) may be linked to cellulose (Cosgrove, 2014). Assuming a pectins’ role as mechanical tethers between microfibrils (Peaucelle et al., 2012; Wang et al., 2012), the combined increased arabinose observed under -N conditions, its expected impact in CW reinforcement, and the preservation of significant plastic deformation capacity, sounds with the existence of “biomechanical hotspots” as proposed by Park and Cosgrove (2012, 2015). The presence of these regions in the CW agrees with the assumption that wall extensibility depends less of the viscoelasticity of matrix polymers and more of the selective separation between microfibrils at limited CW sites. Moreover, under –N and –P, total sugars shift from more easily extractable CW fractions to fractions more tightly attached to insoluble material (Fernandes et al., 2013; Fernandes, 2015). Hydrolytic enzymes were formerly imagined to play a lead role in CW loosening and in the expansion mechanism (Farkas and Maclachlan, 1988; McDougall and Fry, 1990). Although clear-cut evidences of their activity in breaking crystalline cellulose glycosidic bonds are still missing (Vissenberg et al., 2001; Cosgrove, 2005), a contribution to CW loosening, turning it more pliable for expansion, is recognized (Cosgrove, 2005).

In summary, under conditions of cellulose impairment, CW integrity may be maintained through more XyG and biomechanical hotspots and reinforced arabinan-rich linkage points and modified structure of pectins that are bonded to 1,4-glycan polymers, acting as load-bearing components. In Arabidopsis, it was demonstrated that xylan chains were attached to RG-I chains and mediate the adsorption of mucilage to cellulose microfibrils, suggesting a binding affinity of xylose ramifications on RG-I to a cellulose scaffold (Ralet et al., 2016). Cellulose-pectin interactions occur when pectin was present during cellulose synthesis and the strength of the pectin-calcium gel affects its structure and crystallinity (Lopez-Sanchez et al., 2017). More pectins may also have a role in preventing an increase in intracellular spaces, which are associated with decreased CW stiffness (Pieczywek et al., 2017). These results highlight the importance of assembly order on the properties of cellulose composite networks and support the role of pectin in the mechanics of CWs (Lopez-Sanchez et al., 2017).

Even though the cellulose content remained unaffected, *V. vinifera* tissues developing under –S conditions show similar responses, namely Ca^2+^-complexed pectins, arabinan-rich polymers, extensins, and, interestingly, more affected CW-related genes when compared to –N and –P samples. One can speculate a common regulatory point in the pathways of cellulose impairment and compensatory mechanisms that outcomes the latter while a specificity of –S stress allows overcoming the former.

Finally, it can be concluded that the impact of each mineral on CW composition and structure is mostly specific. As expected, nitrogen effects more extensively while the impacts of sulfur are the less pronounced. Although both elements integrate the composition of amino acids and consequently, of proteins, N occurs is some order of magnitude higher than S, and its scarcity has more dramatic effects on protein synthesis, hampering the compensation by other proteins.

No evidences are available to ascertain the specific point of the cellulose deposition pathway that is affect by –N and –P depletion. In fact, CesAs and some GH9 members’ gene expression suggest that the imposed conditions could have affected correct processing or assemblage rather than cellulose synthesis. Future work should focus the functional role of GH9C, but also XTH with hydrolase activity members, in response to these and other abiotic stresses. Understanding if cellulose is less redundant in the CW than pectins, as a heterogeneous family of polymers, should be also the subject of further investigation.

Recognizing that nutrient deficiency triggers responses that compromise the CW, from an agronomic point of view this evidence hastens widening the research to field experiments. As sustainable viticulture practices currently recommend deficit irrigation, which is known to lead to N deficit, and P environment limitation is foreseen in the next decades, predicting changes in plant development to produce biomass and yield would provide valuable information for growers and breeders.

## Author Contributions

LG and SA wrote the MS. JF is the first author of CW grapevine papers and Ph.D. thesis in which this review is based. All authors approved the final version to be published.

## Conflict of Interest Statement

The authors declare that the research was conducted in the absence of any commercial or financial relationships that could be construed as a potential conflict of interest.
