# Supplementary material for: How the Depletion in Mineral Major Elements Affects Grapevine (Vitis vinifera L.) Primary Cell Wall
Source: Front Plant Sci. 2017 Aug 21;8:1439. doi: 10.3389/fpls.2017.01439 (PMC5566972; doi:10.3389/fpls.2017.01439)
Supplement: Supplementary file 1 [file Table_1.PDF]

Supplementary material

Table 1. NCBI accession number of *V. vinifera* CW-associated genes responsive to major mineral elements

Responsive CW-associated gene	NCBI accession number
VviCesA1	XM_010650350
VviCesA2	XM_002277677
VviCesA3	XM_002278961
VviCesA6	XM_010647140
VviCesA8	XM_019222519
VviGH9C2	XM_002270844
VviEXPA5	AM442310
VviEXPA6	XM_002283494
VviEXPA11	XM_002266589
VviEXPA19	XM_002285855
VviEXPA20	XM_002282205
VviXTH2	XM_002285104
VviXTH4	XM_002268672
VviXTH8	XM_002264959
VviXTH14	XM_002274368
VviXTH15	XM_002273706
VviXTH31	XM_002275826
VviXTH32	XM_002269249
VviPMEI2	XM_002263992
VviPMEI3	XM_002263955
VviPMEI4	XM_002263242
VviPMEI5	XM_002284748
VviPME1.4	XM_002277166
VviPME1.11	XM_010651086
VviPME1.17	XM_002273391
VviPME1.18	XM_002266285
VviPME1.19	XM_002267806
VviPME2.7	XM_002274964
VviPME2.11	XM_010663356
VviPME2.14	XM_003631904
VviPME2.20	XM_002268456
